# 1172. SARS-CoV-2 Vaccine Hesitancy in Caregivers of Hospitalized Children

**DOI:** 10.1093/ofid/ofab466.1365

**Published:** 2021-12-04

**Authors:** Marisa Orbea, Rachel Cunningham, C Mary Healy, Julie A Boom, Claire Bocchini

**Affiliations:** 1 Baylor College of Medicine, Texas Children’s Hospital, Houston, Texas; 2 Texas Children’s Hospital, Houston, Texas; 3 Baylor College of Medicine, Houston, TX

## Abstract

**Background:**

SARS-CoV-2 vaccine hesitancy (VH) is hindering nationwide vaccination efforts; little is known about caregiver SARS-CoV-2 vaccine acceptance for children. We aimed to identify associations with SARS-CoV-2 VH in caregivers of hospitalized children.

**Methods:**

We conducted a prospective cross-sectional survey in English and Spanish of caregiver COVID-19 knowledge, attitudes, behaviors, and associated VH among hospitalized children 6 months - 18 years at a large pediatric medical institution. Parents were approached daily, averaging 4-5 days/week, from 12/8/2020--4/5/2021. VH was assessed using the Parent Attitudes about Childhood Vaccines (PACV) survey; PACV score ≥50 denoted VH. Descriptive statistics and multivariable logistic regression were used. Responses were categorized.

**Results:**

295/307 (96%) of approached caregivers enrolled; 79% were ≥ 30 years, 68% were married/ living with a partner, and 57% had at least some college. 36% identified as white, 19% Black, and 46% Hispanic/ Latino. 53% of caregiver children had public insurance. 91% of caregivers self-reported their children were up to date with routine vaccines. 17% of caregivers were vaccine-hesitant overall. 50% of caregivers were willing to receive COVID-19 vaccine themselves. Figure 1 shows intention to vaccinate their child by PACV score.

65% knew someone who was hospitalized for COVID-19. 67% were scared of their child getting COVID-19. However, 49% were scared of their child getting the vaccine, 28% did not want to vaccinate their child and 27% were neutral in the intention to vaccinate their child. Caregivers who did not intend to vaccinate their child were more likely to be Black (27% vs. 16%, p=0.04) and less likely to be Hispanic/ Latino (33% vs. 49%, p=0.02). Table 1 shows attitudes, beliefs, and behaviors surrounding the COVID-19 pandemic and vaccine in caregivers who did or did not intend to vaccinate their child.

Figure 1

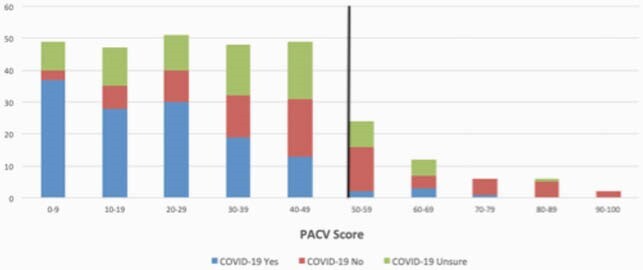

COVID-19 vaccine uptake by PACV score

Table 1

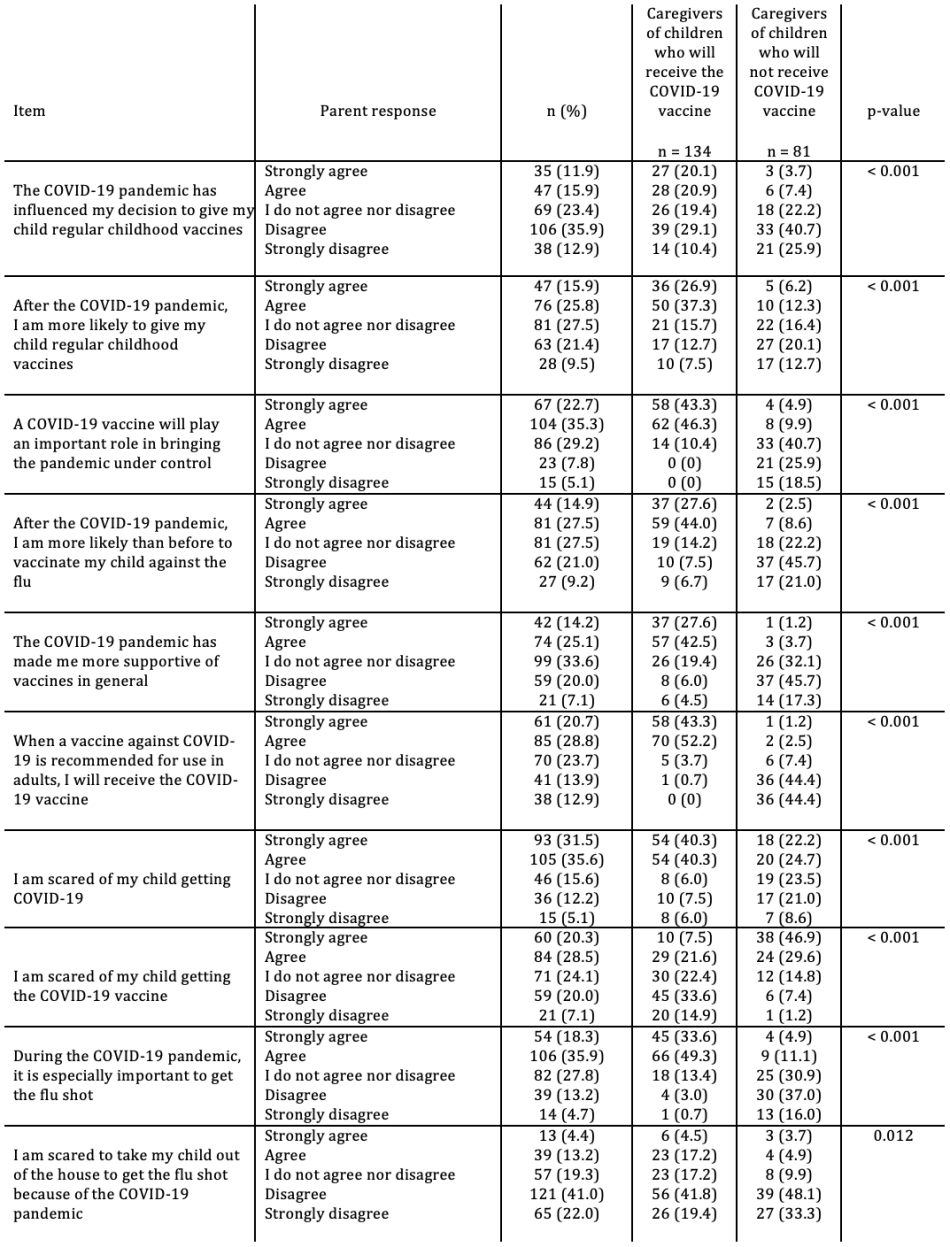

Caregiver attitudes, beliefs, and behaviors surrounding the COVID-19 pandemic and the COVID-19 vaccine

**Conclusion:**

The majority of caregivers believe that SARS-CoV-2 vaccine will help control the pandemic, but less than half plan to vaccinate their children. A quarter of caregivers expressed uncertainty regarding the vaccine and therefore may be amenable to education and discussion. COVID-19 VH is different from VH towards routine vaccinations. More research is needed to address COVID-19 specific VH.

**Disclosures:**

**C. Mary Healy, MD**, **Dexcom** (Shareholder)**Intuitive** (Shareholder)**Quidel Corporation** (Shareholder)**Up to Date** (Other Financial or Material Support, Honorarium)**Vapotherm** (Shareholder)

